# Simple Erosion Narrowing Score of the hands as a predictor of cervical spine subluxation in rheumatoid arthritis

**DOI:** 10.4102/sajr.v24i1.1876

**Published:** 2020-07-27

**Authors:** Eric Gous, Mahmood M.T.M. Ally, Pieter W.A. Meyer, Farhana E. Suleman

**Affiliations:** 1 Department of Radiology, Faculty of Health Sciences, University of Pretoria, Pretoria, South Africa; 2 Department of Rheumatology, Faculty of Health Sciences, University of Pretoria, Pretoria, South Africa; 3 Department of Medical Immunology, Faculty of Health Sciences, University of Pretoria, Pretoria, South Africa

**Keywords:** rheumatoid arthritis, SENS, cervical spine, subluxations, hands

## Abstract

**Background:**

Involvement of the cervical spine is common in patients with rheumatoid arthritis and can lead to devastating or even fatal consequences. Currently no guidelines exist as to whether radiographs of the cervical spine should be included in follow-up visits.

**Objectives:**

To determine whether the Simple Erosion Narrowing Score (SENS) of the hands correlate with the presence of cervical spine subluxation in patients with rheumatoid arthritis.

**Method:**

This was a retrospective, observational, cross-sectional study. A total of 56 rheumatoid arthritis patients with hand radiographs and lateral radiographs of the cervical spine were evaluated. The SENS of the hands and the presence of cervical spine subluxation were compared. The SENS of the hands was correlated with the prevalence of cervical spine subluxation, as was the erosion and joint space narrowing scores of the hands.

**Results:**

A correlation between the SENS of the hands and the prevalence of cervical spine subluxation was confirmed. A higher prevalence of cervical spine subluxation correlated with an increase in the SENS of the hands (*p* = 0.0002). The erosion and joint space narrowing scores of the hands also correlated with the prevalence of cervical spine subluxation (*p* = 0.0001).

**Conclusion:**

This study confirmed that a correlation exists between cervical spine subluxation, peripheral joint space erosions and joint space narrowing in patients with rheumatoid arthritis and SENS may therefore be used as a predictor of cervical spine disease.

## Introduction

Rheumatoid arthritis (RA) is an autoimmune mediated disease characterised by chronic inflammation of synovial joints and involvement of the cervical spine.^1,2,3,4,5,6,7^ The chronic inflammation of RA leads to damage in cartilage and subchondral bone which leads to joint destruction.^8,9^ Cervical spine damage due to RA may give rise to myelopathy and sudden death due to spinal cord and brain stem compression.^10^ The most common and notable patterns of cervical spine instability in RA include: atlantoaxial subluxation; cranial settling; sub-axial subluxation; or a combination of the above.^2,4,5,6,7,10^

Atlantoaxial subluxation occurs as the result of an incompetent transverse ligament or erosion of the dens. It usually occurs in the anterior direction but may be in a posterior or lateral direction. Cranial settling decreases the vertical distance between the brainstem and the dens and is caused by erosion in the occipito-atlanto articulation, the atlantoaxial articulation or both. Sub-axial subluxation is caused by facet joint erosion and ligament incompetence and can occur at multiple levels.^4,5,6^

The association between peripheral joint destruction and cervical spine subluxation in RA is known,^2^ but only one previous study has correlated the Simple Erosion Narrowing Score (SENS) of both the hands and feet with the prevalence of cervical spine subluxation.^11^ Our study is the first to correlate SENS of the hands only with cervical spine subluxation.

Scoring methods are used to evaluate the severity and monitor the progression of RA.^8,12^ Simple Erosion Narrowing Score is a radiological scoring method for peripheral joints based on the presence or absence of erosions and joint space narrowing.^8,9,13^

This study evaluated the SENS of the hands as a predictor of cervical spine subluxation in patients with RA and may provide clinicians with a rapid method of predicting cervical spine subluxation in RA patients.^8,9^ Currently, no guidelines exist as to whether radiographs of the cervical spine should be performed on a regular basis,^10^ but predicting cervical spine subluxation with the SENS of the hands may serve as a guideline, which might lead to decreased radiation exposure for patients, more efficient utilisation of available resources, as well as cost limitation.

The aim of the study was to determine whether the SENS of the hands can be used to predict the presence of cervical spine subluxation in patients with RA and the objectives were to assess whether there is correlation between the SENS of the hands and the prevalence of cervical spine subluxation (atlantoaxial subluxation/cranial settling/sub-axial subluxation) in patients with RA, as well as to develop a guideline based on the SENS of the hands in patients with RA for radiological investigation of the cervical spine.

## Methods

This was a retrospective, observational, cross-sectional study set in the Department of Radiology and Department of Rheumatology, Steve Biko Academic Hospital, Pretoria.

Radiographs of 56 RA patients at Steve Biko Academic Hospital’s rheumatology clinic were evaluated. To be eligible, radiographs needed to include at least one set of Anterior-Posterior (AP) radiographs of both hands, as well as lateral radiographs of the cervical spine in the neutral, flexion and extension positions taken on the same day.

**FIGURE 1:**
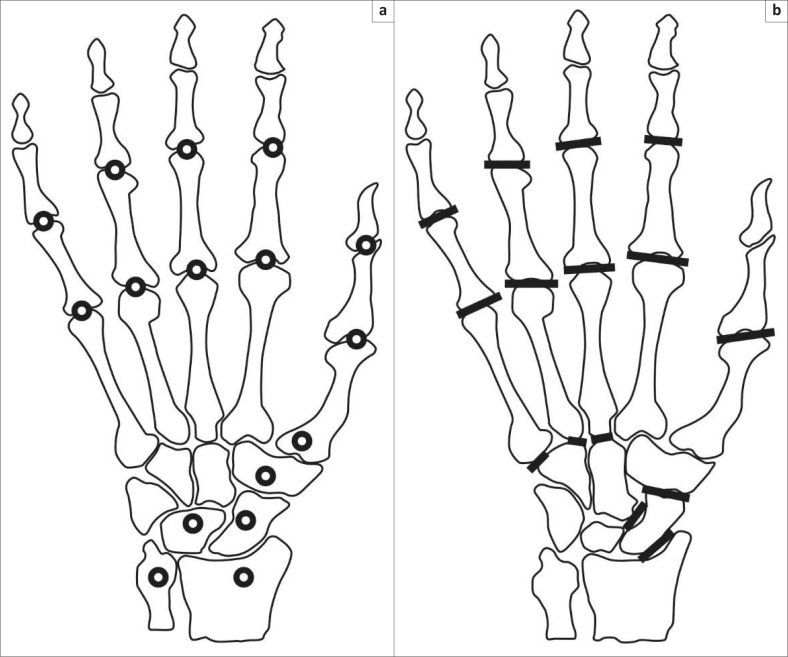
Diagram illustrating joints of the hand assessed for (a) erosions and (b) joint space narrowing for Simple Erosion Narrowing Score.

The Simple Erosion Narrowing Score is used to score radiographs of both hands and feet,^8,9^ but in this study, only the SENS of the hands was used.^8,9,13^ A total of 32 joints were scored for erosions and 30 for joint space narrowing (Figure 1). A joint is scored 1 point if there is erosion at least at one location and another 1 point if joint space narrowing is present. The maximum erosion score was 32, the maximum joint space narrowing score was 30 and the maximum total SENS was 62 for the hands.^8,9,13^

Lateral radiographs of the cervical spine in the neutral, flexion and extension positions were evaluated to determine the presence of cervical spine subluxation. Atlantoaxial subluxation was diagnosed if the anterior atlanto-dental interval (AADI) was > 3 mm on any of the lateral cervical spine radiographs. For the evaluation of cranial settling, the Redlund-Johnell measurement was used, which measures the distance between the inferior border of the C2 vertebral body and a line drawn from the posterior tip of the hard palate to the inferior cortical margin of the occiput (known as the McGregor Line). A result of less than 34 mm in males and less than 29 mm in females was used to diagnose cranial settling.^4,5^ A diagnosis of sub-axial subluxation was made if a vertebra was displaced > 3 mm anteriorly or posteriorly in relation to the next vertebra when measured from the posterior line of the vertebral bodies in flexion or extension.^7^

Radiographs were evaluated by a single radiologist; a senior registrar in the department who had completed his Fellowship of the College of Diagnostic Radiologists of South Africa (FC Rad Diag SA) part 2 exam and had an interest in musculoskeletal radiology. Separate diagrams for scoring erosions and joint space narrowing of the hands were used to determine the SENS of each patient and the result was combined with the cervical spine subluxation measurements on a single chart for each patient.

The prediction equation of interest was a logistic regression function with dependant variable ‘outcome’, i.e. zero if all three types of cervical spine subluxation were absent and one if at least one of the three types of cervical spine subluxations were present. Sample size calculation was based on the number of Events Per Variable (the sample size is adequate when the Events Per Variable [EPV] > 5.16). Three prediction variables were evaluated with the SENS (hands along with sex and age). To reach the expected proportion of cervical spine subluxation of 32.5%, the required sample size was 47 in which the number of events were more than 15 (3 × 5).^14^

Data summary included mean, standard deviation, range and 95% confidence intervals (CI). For discrete data, frequencies, percentage, cross-tabulation and 95% CI were used. Intra-reader reliability was derived as the intra-class correlation coefficient (ICC) from a random-effects maximum likelihood regression analysis. The relationship between the SENS of the hands and the cervical spine subluxation outcome (present/absent) was assessed using diagnostic statistics (sensitivity, specificity, positive predictive and negative predictive values). The main aim of the study was to evaluate the relationship between the SENS of the hands and cervical spine subluxation, but the erosion and joint space narrowing scores of the hands and cervical spine subluxation were also assessed separately. A post-hoc logistic regression analysis was employed to derive the diagnostic statistics.

### Ethical consideration

This was a retrospective, observational, cross-sectional study. Ethics approval was obtained from the Ethics committee of the Health Sciences Faculty at the University of Pretoria (Ethics number 69/2016). Custodian consent was obtained from Steve Biko Academic Hospital’s chief executive officer to access patient files.

## Results

A total of 56 patients with RA at Steve Biko Academic Hospital’s rheumatology clinic were evaluated. The SENS of the hands was determined on AP radiographs of the hands and the presence of cervical spine subluxation was determined on lateral radiographs of the cervical spine in the neutral-, flexion- and extension positions. The SENS of the hands was correlated with the prevalence of cervical spine subluxation to determine whether it can be used as a possible predictor of cervical spine subluxation. A random-effects maximum likelihood regression analysis was used to determine an ICC, which is used as a measure of intra-reader reliability. A result of 0.91 was obtained, which shows excellent repeatability (maximum ICC = 1).

Descriptive statistics for continuous variables are summarised in Table 1. Proportions, as well as 95% CI for binary variables are summarised in Table 2.

**TABLE 1:** Descriptive statistics for continuous variables.

Variable	Descriptive statistics for continuous variables					
	Mean	SD	Median	Range	Geometric mean†	95% CI
Age	54.9	13.58	57.0	19–79	-	51.3; 58.6
AADI	2.8	1.93	2.0	1–11	2.31	1.99; 2.69
Erosion score	5.2	6.77	3.0	0–26	2.56	1.67; 3.74
Narrowing score	5.4	7.83	2.0	0–30	2.21	1.34; 3.40
SENS	10.6	13.97	5.0	0–55	4.49	2.89; 6.76
RJM (entire study population)	36.5	5.86	36.5	19–57	-	34.93; 38.07
Male RJM	43.0	6.07	41.0	38–57	-	37.92; 48.08
Female RJM	35.4	5.13	35.5	19–44	-	33.93; 36.91

AADI, anterior atlantodens interval; SENS, Simple Erosion Narrowing Score; RJM, Redlund-Johnell measurement; CI, confidence interval; SD, standard deviation.

† , Where distribution was skew, that is, mean and median different in relation to scale of measurement, the geometric mean should be interpreted.

**TABLE 2:** Proportions, as well as 95% confidence interval for binary variables.

Variable	Proportions and 95% CI for binary variables		
	Proportion (*N* = 56)		95% CI
	%	*n*	
Female	85.7	48	73.78; 93.62
Atlantoaxial subluxation present	19.6	11	10.24; 32.43
Cranial settling present	7.1	4	1.98; 17.29
Sub-axial subluxation present	10.7	6	4.04; 21.88
Total subluxations present (single subluxation/combination of subluxations)	28.6	16	17.30; 42.21

CI, confidence interval.

Cervical spine subluxation (a single subluxation or a combination of subluxations) was present in a total of 16 (28.6%) of the 56 patients. Atlantoaxial subluxation, cranial settling and sub-axial subluxation was present in 11 (19.6%), 4 (7.1%) and 6 (10.7%) patients respectively. In the 16 patients with cervical spine subluxation, atlantoaxial subluxation was present in 6 (37.5%) patients, cranial settling in 2 (12.5%) patients, sub-axial subluxation in 3 (18.8%) patients and a combination of subluxations in 5 (31.3%) patients.

**FIGURE 2:**
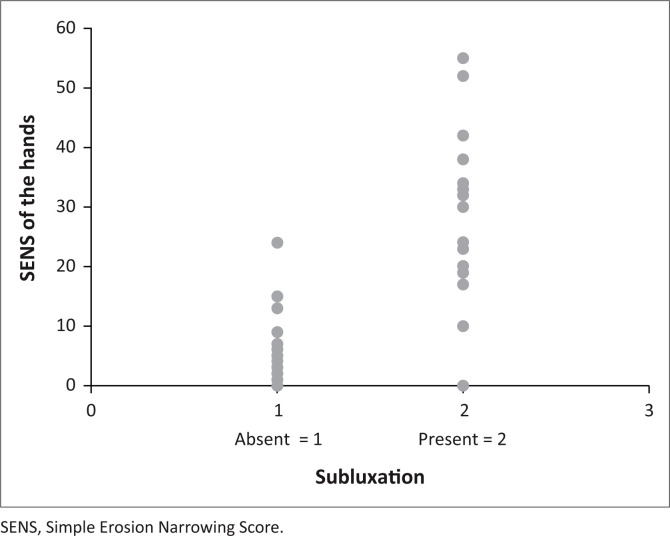
Correlation between the Simple Erosion Narrowing Score of the hands and the prevalence of cervical spine subluxation.

**FIGURE 3:**
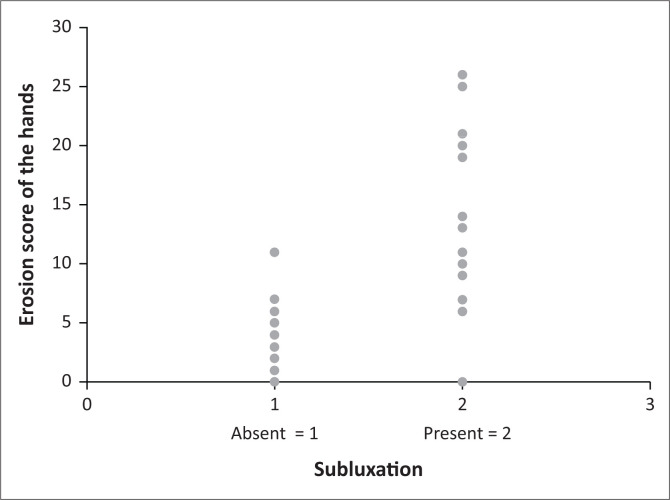
Correlation between the erosion score of the hands and the prevalence of cervical spine subluxation.

**FIGURE 4:**
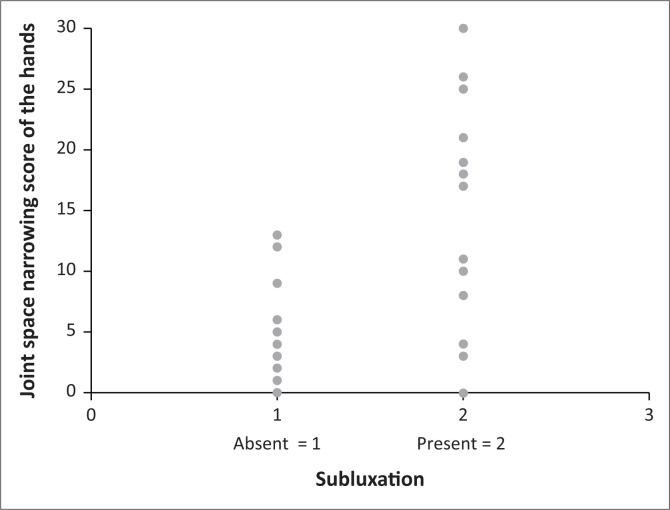
Correlation between the joint space narrowing score of the hands and the prevalence of cervical spine subluxation.

A correlation between the SENS of the hands and cervical spine subluxation (*p* = 0.0002) was found and is demonstrated in Figure 2. A higher prevalence of cervical spine subluxation was found with an increase in the SENS. The same result was obtained for the erosion and joint space narrowing scores of the hands when correlated with the prevalence of cervical spine subluxation separately (*p* = 0001). The correlations between the increase in the SENS, erosion, and joint space narrowing scores of the hands and the prevalence of cervical spine subluxation are demonstrated in Figures 2, 3 and 4 respectively.

Study results using the SENS of the hands as a predictor of cervical spine subluxation are summarised in Table 3.

**TABLE 3:** Simplified erosion narrowing score of hands, presence of erosions and joint space narrowing as predictors of cervical spine subluxation.

Indicator	Sensitivity		Specificity		LLR (+)		LLR (-)		Odds Ratio		PPV		NPV		LLR χ^2^ (*P*)
	%	CI	%	CI	n	CI	n	CI	n	CI	n	CI	n	CI	
SENS	82	57–96	95	83–99	15	4–63	0.19	0.07–0.52	86	14–523	88	62–98	93	80–98	0.0002
Erosions	82	57–96	95	83–99	15	4–63	0.19	0.07–0.52	86	14–523	88	62–98	93	80–98	0.0001
JSN	59	36–79	91	76–98	7	2–21	0.45	0.27–0.75	15	4–60	81	54–96	78	62–89	0.0001

SENS, Simplified erosion narrowing score; LLR (−), Negative likelihood ratio; NPV, Negative predictive Value; JSN, Joint space narrowing; LLR(+), Positive likelihood ratio; PPV, Positive predictive value; LLRχ2, Likelihood ration Chi-square; CI, confidence interval.

The SENS of the hands serves as a relatively good predictor of cervical spine subluxation in patients with RA. Out of the 16 patients in which cervical spine subluxation was present, 14 had a SENS of the hands > 9 (true positives) and 2 had a SENS of the hands ≤ 9 (false negatives). Of the 40 patients with no cervical spine subluxation, 3 had a SENS of the hands > 9 (false positives) and 37 had a SENS of the hands ≤ 9 (true negatives). A SENS of the hands > 9 as a predictor of cervical spine subluxation in this study population demonstrated a sensitivity of 87.5 %, specificity of 92.5 %, positive predictive value (PPV) of 82.4 % and negative predictive value (NPV) of 94.9 %.

Study results using the erosion- and joint space narrowing scores of the hands as predictors of cervical spine subluxation, as well as the sensitivity, specificity, PPV and NPV of the SENS, erosion and joint space narrowing scores of the hands as predictors of cervical spine subluxation are summarised in Table 3.

## Discussion

The patients with cervical spine subluxation generally had higher SENS scores, and erosion and joint space narrowing scores of the hands, when compared to patients where cervical spine subluxation was absent. The majority of patients with a relatively low SENS of the hands did not have cervical spine subluxation, with the exception of a small percentage of patients with a low SENS who presented with cervical spine subluxation. In general, a higher SENS correlated with a higher prevalence of cervical spine subluxation, but a low SENS did not necessarily exclude cervical spine subluxation.

This study confirmed the correlation between SENS of the hands and the prevalence of cervical spine subluxation in patients with RA (*p* = 0.0002). This is in accordance with other studies.^2,3,7,10,11,12,13,14,15^ The other objective of this study was to develop a guideline regarding the radiological investigation of the cervical spine based on the SENS in patients with RA. The purpose of such a guideline would be to take only an image of the cervical spine in patients where cervical spine subluxation is more likely, based on the SENS of the hands. Diagnostic statistics to predict cervical spine subluxation were found to be most optimal for a SENS of the hands > 9 and absent where the SENS of the hands was ≤ 9. If this guideline is to be implemented in clinical practice, the cervical spine would only be imaged in those patients where the SENS of the hands are > 9. However, a SENS of the hands > 9 as a predictor of cervical spine subluxation in this study population produced 2 false negatives, thus slightly lowering the sensitivity to 87.5 % and the PPV to 82.4 %. This means that 2 out of the 16 cervical spine subluxations would not be diagnosed if imaging based on the SENS of the hands alone were to be used for diagnosing cervical spine subluxation. The 2 patients (false negatives) in this study with a low SENS of the hands (0) and cervical spine subluxation both had atlantoaxial subluxation.

The erosion- and joint space narrowing scores of the hands as predictors of cervical spine subluxation in patients with RA were also evaluated separately. While a higher prevalence of cervical spine subluxation was found with an increase in the SENS, the same result was obtained for the erosion and joint space narrowing scores of the hands when correlated with the prevalence of cervical spine subluxation separately (*p* = 0.0001). For the erosion score of the hands, diagnostic statistics for predicting cervical spine subluxation were found to be most optimal for a score > 5, that is, cervical spine subluxation present where the erosion score of the hands was > 5 and absent where the erosion score of the hands was ≤ 5. For the joint space narrowing score of the hands, diagnostic statistics for predicting cervical spine subluxation were found to be most optimal for a score of > 3, that is, cervical spine subluxation present where the joint space narrowing score of the hands was > 3 and absent where the joint space narrowing score of the hands was ≤ 3.

The erosion score of the hands as a predictor of cervical spine subluxation was equally effective compared to the SENS with identical sensitivity, specificity, PPV and NPV. The erosion score of the hands might thus serve as an easier and quicker but equally effective predictor of cervical spine subluxation than the SENS in patients with RA. The joint space narrowing score was not as effective in predicting cervical spine subluxation as the SENS or the erosion score with a lower sensitivity, specificity, PPV and NPV.

## Conclusion

This study confirmed a correlation between the SENS of the hands and the prevalence of cervical spine subluxation in patients with RA. A higher prevalence of cervical spine subluxation was found with an increase in the SENS. The same results were obtained with the erosion and joint space narrowing scores of the hands when correlated with the prevalence of cervical spine subluxation separately. The SENS of the hands correlates with the prevalence of cervical spine subluxation in patients with RA, and may be used as a relatively reliable predictor of cervical spine subluxation in patients with RA, but SENS alone should not be the only predictive factor taken into account when making decisions regarding the imaging of the cervical spine in patients with RA. Other predictive factors of cervical spine subluxation such as biochemical markers, clinical features, patient demographics and treatment in conjunction with the SENS of the hands should be considered as well.
